# Supplementary figures and images for: Gal3 Plays a Deleterious Role in a Mouse Model of Endotoxemia
Source: Int J Mol Sci. 2022 Jan 21;23(3):1170. doi: 10.3390/ijms23031170 (PMC8835800; doi:10.3390/ijms23031170)

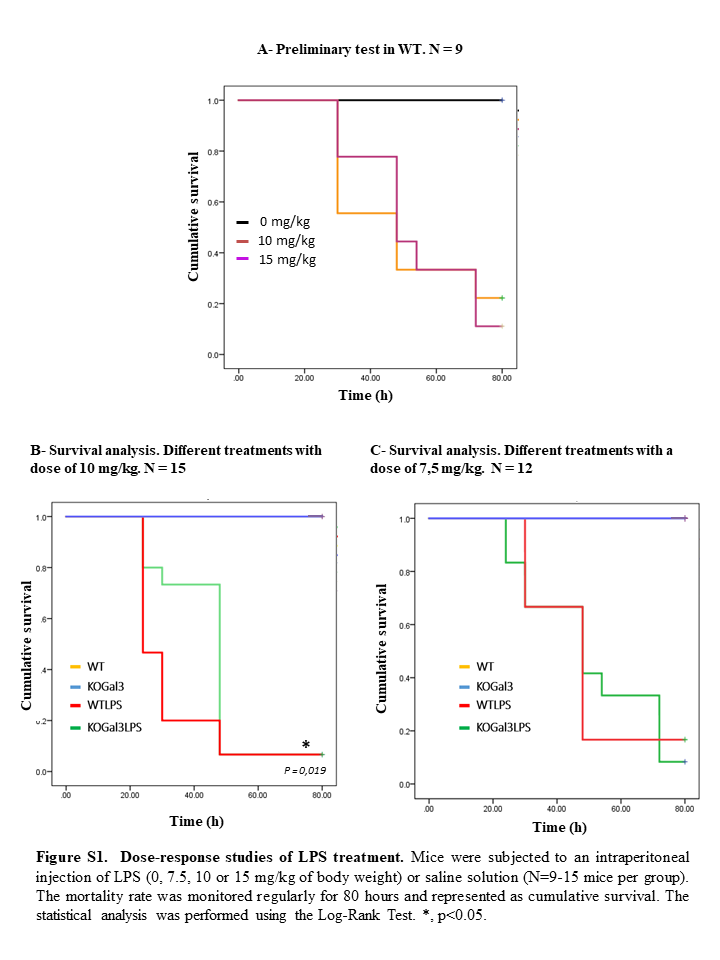

Supplement: Supplementary file 1 [file ijms-23-01170-s001.zip › Figure S1.TIF]

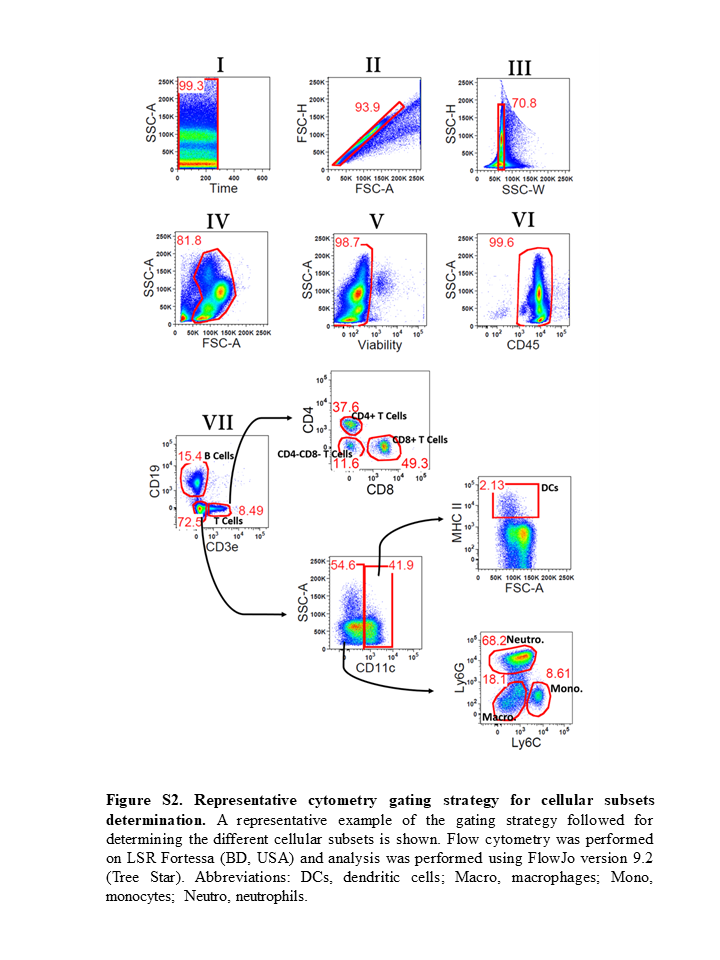

Supplement: Supplementary file 1 [file ijms-23-01170-s001.zip › Figure S2.TIF]

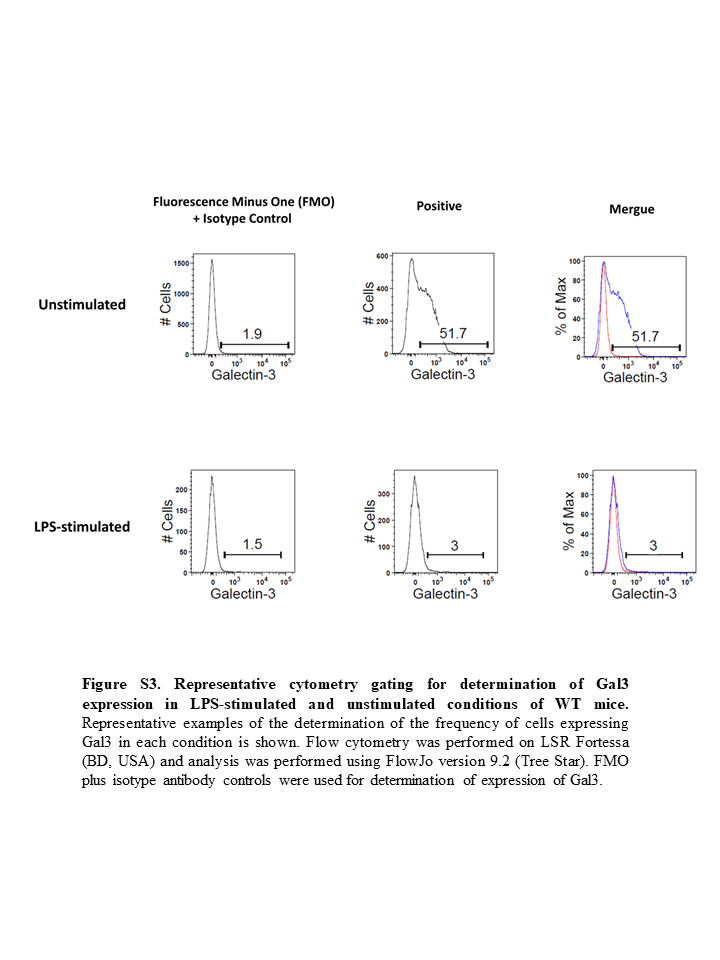

Supplement: Supplementary file 1 [file ijms-23-01170-s001.zip › Figure S3.TIF]

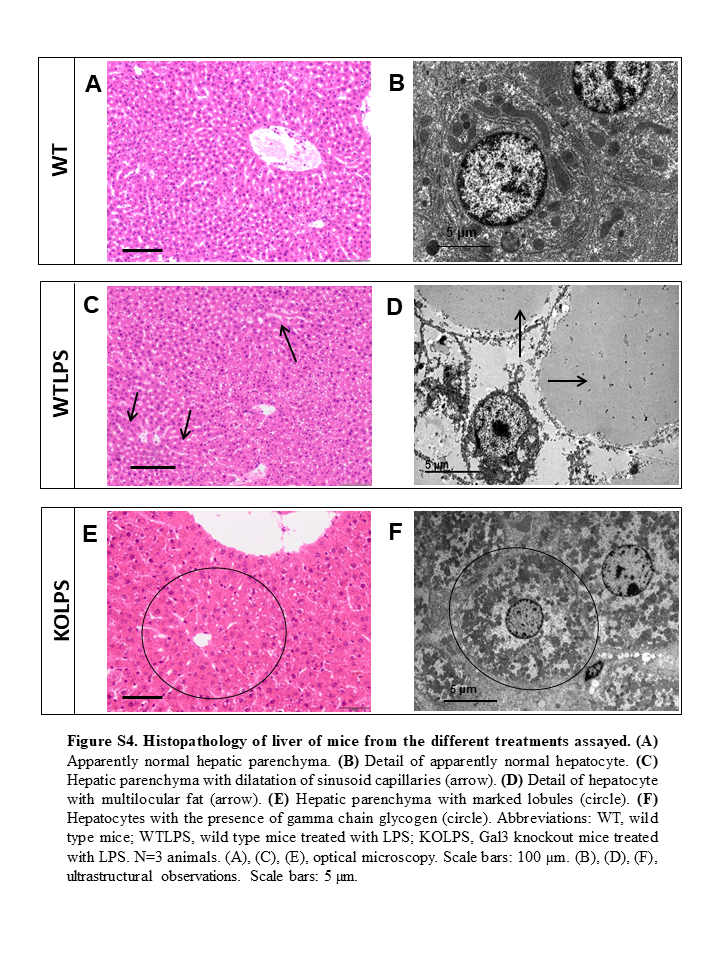

Supplement: Supplementary file 1 [file ijms-23-01170-s001.zip › Figure S4.TIF]

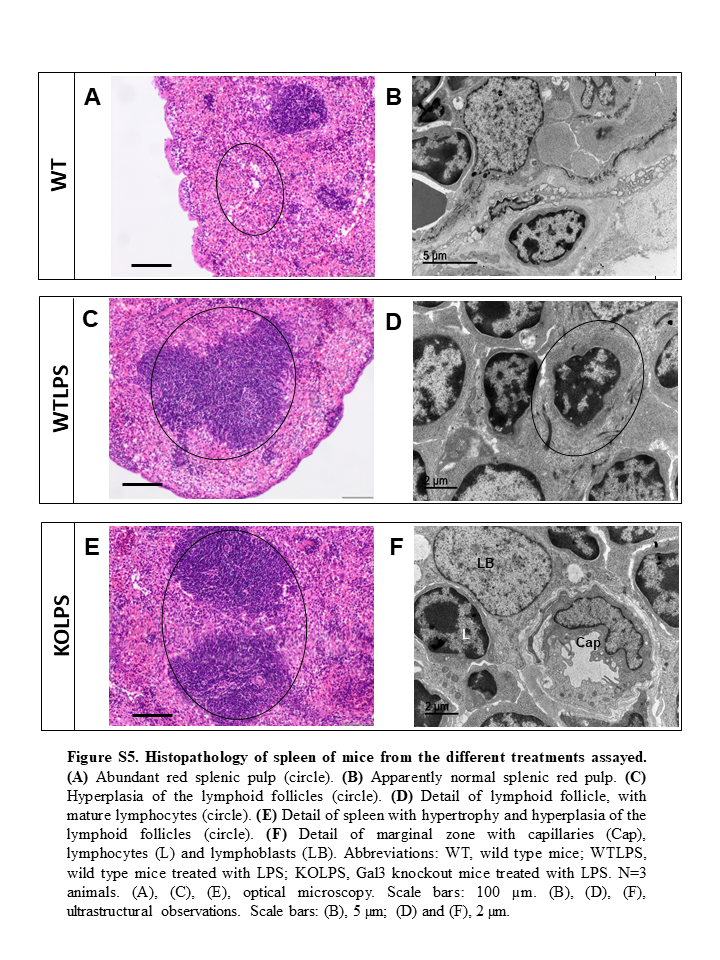

Supplement: Supplementary file 1 [file ijms-23-01170-s001.zip › Figure S5.TIF]

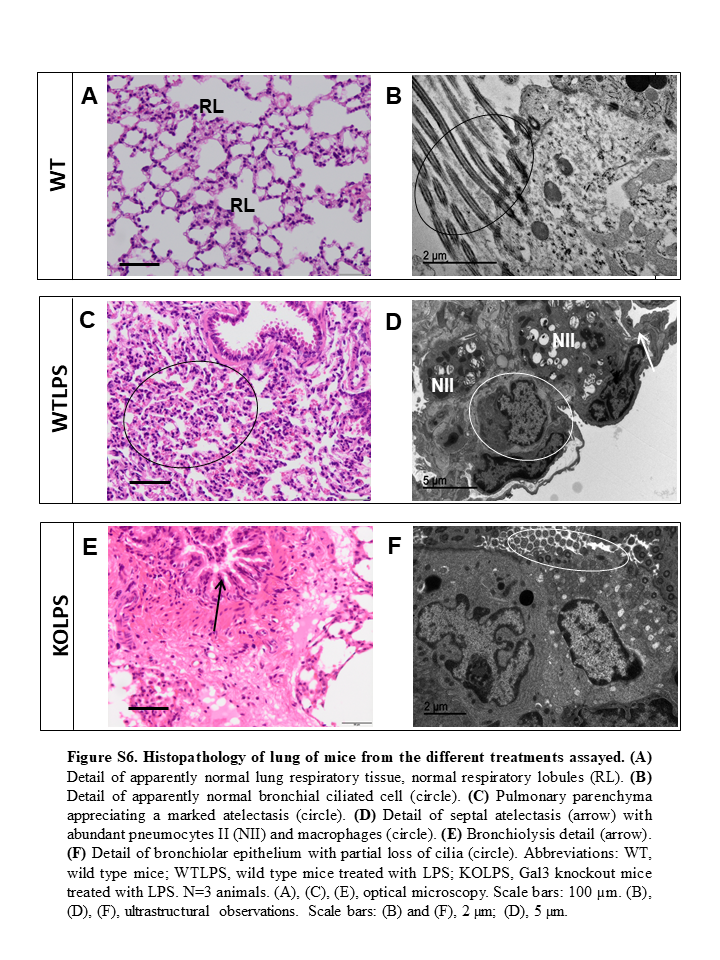

Supplement: Supplementary file 1 [file ijms-23-01170-s001.zip › Figure S6.TIF]

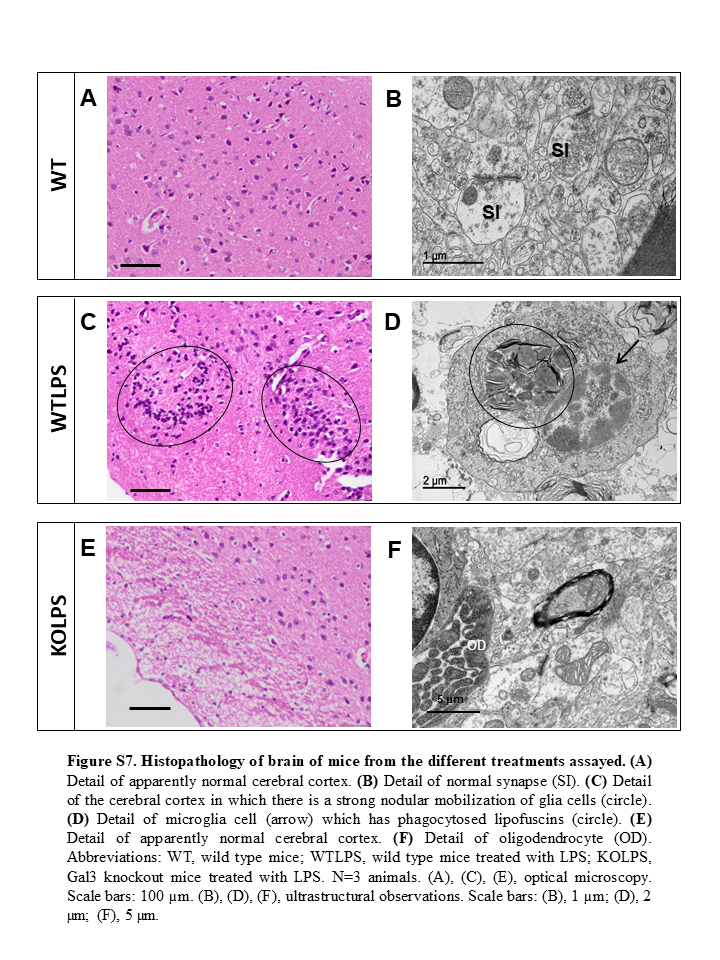

Supplement: Supplementary file 1 [file ijms-23-01170-s001.zip › Figure S7.TIF]

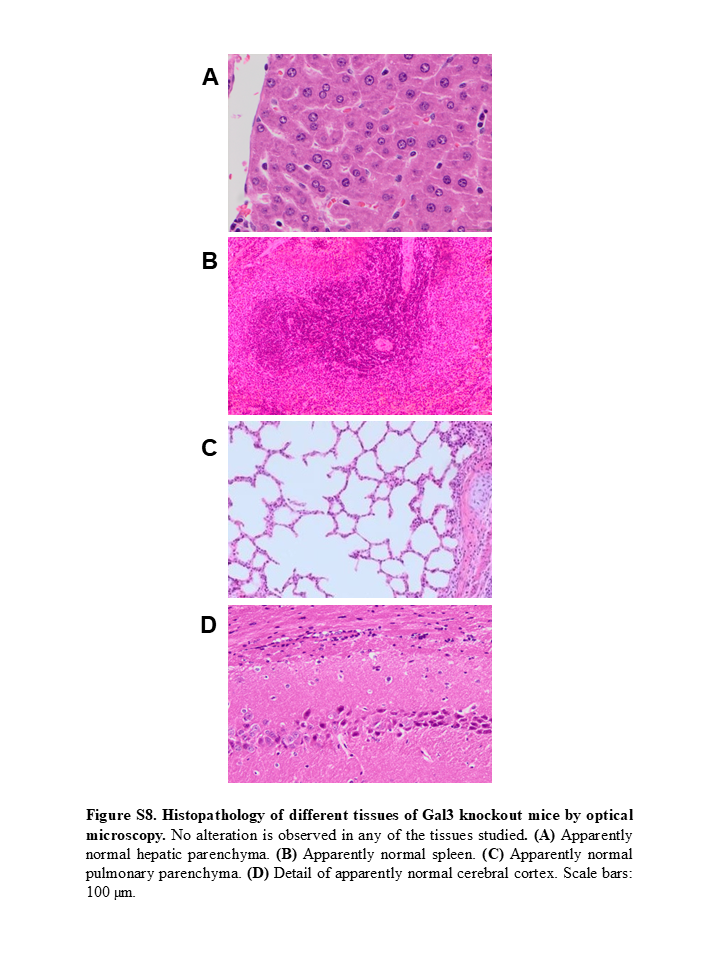

Supplement: Supplementary file 1 [file ijms-23-01170-s001.zip › Figure S8.TIF]
